# Decellularized bovine ovarian niche restored the function of cumulus and endothelial cells

**DOI:** 10.1186/s13104-022-06233-7

**Published:** 2022-11-08

**Authors:** Farhad Amjadi, Rahim Beheshti, Fatemeh Sokouti Nasimi, Ayla Hassani, Reza Shirazi, Amin Tamadon, Reza Rahbarghazi, Mahdi Mahdipour

**Affiliations:** 1 Department of Veterinary Science, Islamic Azad University Shabestar Branch, 5381637181 Shabestar, Iran; 2 Student Research Committee, Tabriz University of Medical Sciences, Tabriz, Iran; 3 Stem Cell Research Center, Tabriz University of Medical Sciences, 5165665811 Tabriz, Iran; 4 Stem Cell and Tissue Engineering Research Laboratory, Sahand University of Technology, 51335-1996 Tabriz, Iran; 5 Chemical Engineering Faculty, Sahand University of Technology, 51335-1996 Tabriz, Iran; 6 Department of Anatomy, School of Medical Sciences, Medicine & Health, UNSW Sydney, Sydney, Australia; 7 Percia Vista R&D Co. Shiraz, Shiraz, Iran; 8 Department of Applied Cell Sciences, Faculty of Advanced Medical Sciences, Tabriz University of Medical Sciences, 5166653431 Tabriz, Iran; 9 Department of Reproductive Biology, Faculty of Advanced Medical Sciences, Tabriz University of Medical Sciences, 5166653431 Tabriz, Iran

**Keywords:** Bovine ovary, Decellularization, Recellularization, Endothelial cells, Cumulus cells

## Abstract

**Objective:**

Recently, the decellularization technique is introduced as one of the tissue engineering procedures for the treatment of various deficiencies. Here, we aimed to assess the dynamic activity of CCs and HUVECs within decellularized bovine ovarian tissue transplanted subcutaneously in rats. Ovarian tissue was decellularized using a cocktail consisting of different chemicals, and the efficiency of decellularization was assessed using hematoxylin-eosin and DAPI staining. The cell survival was evaluated using an LDH leakage assay. Thereafter, decellularized samples were recellularized using HUVECs and CCs, encapsulated inside alginate (1.2%)-gelatin, (1%) hydrogel, and transplanted subcutaneously to rats. The existence of CD31- and estrogen-positive cells was assessed using immunohistochemistry staining.

**Results:**

Bright-field imaging and DAPI staining revealed the lack of nuclei with naive matrix structure in ovarian tissue subjected to decellularization protocol. SEM imaging revealed a normal matrix in decellularized ovaries. LDH assay showed a lack of cytotoxicity for CCs after 7-days compared to the control group. Immunohistochemistry staining showed both CD31- and estrogen-positive cells in CCs + HUVECs compared to the CCs group. CD31 cells appeared with flattened morphology aligned with matrix fibers. The existence of estrogen and CD31 positive cells showed the efficiency of decellularized ovarian tissue to restore cellular function and activity.

**Supplementary Information:**

The online version contains supplementary material available at 10.1186/s13104-022-06233-7.

## Introduction

In females, different etiologies such as inability to ovulate, anatomical dysfunction like fallopian tube problems, endometriosis, age-related changes, and other factors contribute to the loss of ovarian tissue function [1, 2]. Tissue engineering and regenerative medicine are new interdisciplinary branches of science that have been considered to overcome various diseases including reproductive disorders [3]. Along with natural tissue-derived ECM, artificial scaffolds are also at the center of attention for tissue regeneration. However, the desired structure and toxicity are the main objections that limit their application in tissue engineering [4]. The decellularization process is a stepwise procedure to create a suitable natural scaffold for cell replacement and subsequently transplantation [3, 5]. Decellularized scaffolds possess in vivo-like micro- and nanostructures with suitable physicomechanical and biological properties for cell bioactivity [6]. Likewise, the stability of the natural 3D structure provides clues for proper regeneration and supports ovarian cells to restore normal function [7]. During the past years, different protocols have been used for tissue decellularization using various detergents [8, 9]. For instance, SDS, peracetic acid, Tween 20, and TritonX-100 are common chemicals used to remove cytoplasmic proteins, genetic material, and cellular and molecular components of antigens while maintaining the ECM [5, 9, 10]. After the completion of the decellularization procedure, both stem cells and mature cell types can be used to restore ovarian tissue function. Despite these advances, promising results have not been achieved yet.

**Fig. 1:**
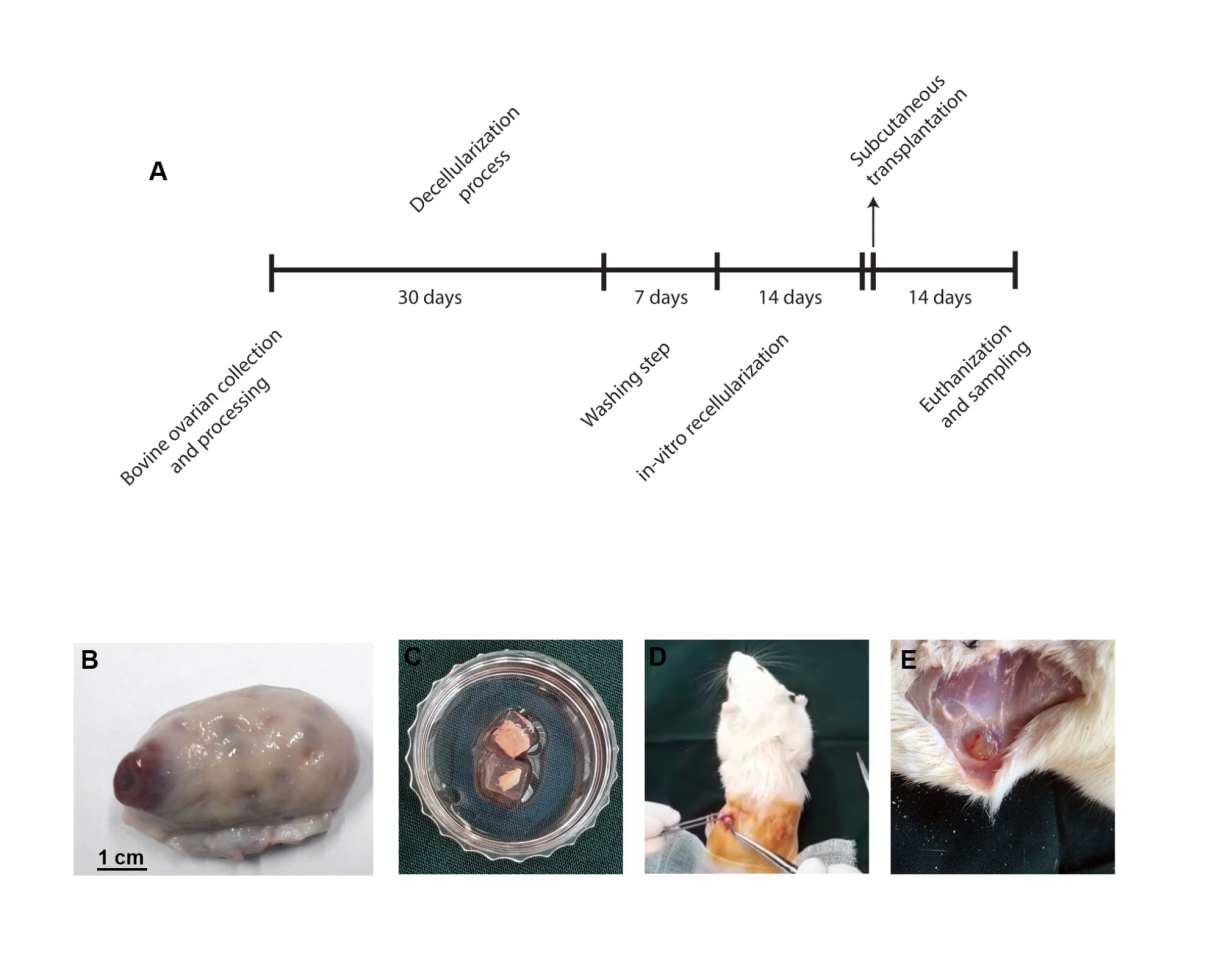
Experimental setup. The general timeline of the investigation (**A**), Bovine ovarian tissue (**B**), decellularized tissue covered by gelatin (**C**), transplantation procedure (**D**), and sampling (**E**)

Alginate is a natural-based polysaccharide and is commonly used for the fabrication of hydrogel for the encapsulation of cells and tissue fragments [11, 12]. Several studies have used ionic cross-linkers such as Ca^2+^, Ba^2+^, and Sr^2+^ for the formation of polymeric networks. The addition of gelatin belonging ECM component can help cells to easily attach the hydrogel [13]. In this experiment, we aimed to assess the regenerative potential of HUVECs and Bovine CCs in the reconstitution of decellularized bovine ovaries in in vitro and in vivo conditions (Fig. 1 A-E). The combination of alginate and gelatin was used as supporting hydrogel for the subcutaneous transplantation of recellularized ovarian tissue.

## Materials and methods

The current study was performed under previously published guidelines [The Animals (Scientific Procedures) Act 1986].

### Sample collection and decellularization

Bovine ovaries were collected from abattoir samples in Tabriz industrial slaughterhouse (Fig. 1B). Ovaries were incubated in 0.9% sodium chloride solution supplemented with 1% penicillin and streptomycin and maintained at 30–35 °C for less than 2 h. The decellularization procedure is described in the online Method and Materials section.

### Confirmation of decellularization procedure

For H&E and DAPI staining, samples were placed in a 10% buffered formalin solution, paraffin-embedded, and sections of 5-µm thick were prepared using microtome instrument. Following deparaffinization, sections were stained for 5 min with either H&E solution or 1 µg/mL DAPI and rinsed with PBS [14]. At the end, sections were evaluated using an Olympus microscope. Besides, tissues were fixed in 2.5% glutaraldehyde solution for 8 h and exposed to various ascending serial concentrations of alcohol (50, 70, 96, and 100%). Following gold sputtering, samples were imaged using a scanning electron microscope (SEM; model: MIRA3 FEG-SEM, Tescan, Czech Republic).

### CCs isolation and expansion

For more data, please refer to the online Method and Materials section.

### LDH assay

For more data, please refer to the online Method and Materials section.

### Recellularization step

The ovarian protein extract was prepared by the incubation of fresh ovarian samples in RIPA buffer (150 mM NaCl, 0.1% SDS, 50 mM Tris-HCl, 2 mM EDTA, and 1% NP-40). To this end, freshly prepared ovarian tissue was washed with PBS, mechanically chopped, and exposed to the protein lysis buffer. The final concentration of protein was determined using a BCA assay (Cat no: 21,072; SMART micro BCA). About 2.5 µg/tissue was used in all groups. Here, cells were allocated into three different groups as follows; Control (decellularized tissues without cells), CCs, and CCs + HUVECs groups. In CCs + HUVECs group, a total of about 30 × 10^4^ cells (per 9.6 cm^2^) with a ratio of 1:1 was used. The cells were collected in 25 µL with protein extract and injected into decellularized samples using a G31 syringe. In the CCs group, about 30 × 10^4^ cells were used with a similar protocol. The samples were kept in culture condition for 14 days.

### Subcutaneous implantation of recellularized ovarian tissues

On the day of tissue transplantation, the recellularized tissues were encapsulated using a mixture of alginate-gelatin. To this end, the 2% alginate (Lot no: 4F30155; Funakoshi) solution was dissolved in CF-KRH solution and sterilized using 70% alcohol under a laminar hood. Also, 10% gelatin solution was prepared and autoclaved. Thereafter, a solution containing 1.25% alginate and 1% gelatin was prepared. Tissue pieces were placed in molds and a polymer solution was poured for casting. 200 mM calcium chloride was added to solidify the polymer solution (Fig. 1 C).

### In vivo assay

On day 15, the presumptive recellularized tissues were transplanted to the rats. All the experiments and protocols were approved by the local ethical committee of the Islamic Azad University of Tabriz (IR.IAU.TABRIZ.REC.1399.124). For this purpose, 6 female rats weighing 250–300 g (8–10 weeks old) were purchased from Med Zist Company-Tehran and kept for 5 days in favorable temperature and nutritional conditions for acclimation. For immune system suppression and to minimize the chance of transplant rejection, rats were injected with 1 dose of 100 mg/Kg/BW Cyclophosphamide (Cat no: 6055-19-2; Sigma-Aldrich). Seven days after immunosuppression, 6 rats were allocated into three different groups (n = 2). To induce general anesthesia, ketamine and xylazine solution (90 mg/kg and 10 mg/kg respectively) were intraperitoneally injected. After induction of anesthesia, the supra flank regions were shaved, disinfected using 70% ethanol and povidone-iodine solution, and cut to a length of 0.5 cm (Fig. 1 C). Then, two tissues encapsulated with alginate-gelatin hydrogel were subcutaneously placed on each side of the flank (Fig. 1D). Finally, the incision site was sutured with an absorbent suture. After 3 to 4 h, all the rats regained consciousness. Two weeks after transplantation, all rats were euthanized according to ethical principles after induction of anesthesia and the transplanted tissues were removed and subjected to histological examinations (Fig. 1E).

### Immunohistochemistry (IHC) assay

Briefly, transplants were fixed in a 10% buffered formalin solution and subsequently embedded in paraffin blocks. Thereafter, 5 μm thick sections were exposed to 3% H_2_O_2_ for 20 min to neutralize endo-peroxidase activity. The procedure was followed by blocking in 1% BSA for 30 min and incubation with CD31 (dilution: 1:300; Cat no: sc-376,764; Santa Cruz Biotechnology Inc.) and estrogen receptor (ER) as the primary antibodies (dilution: 1:300; Cat no: sc- 101,103; Santa Cruz Biotechnology Inc.) at 4°C overnight. After PBS washes, slides were incubated with an HRP-conjugated secondary antibody (Cat no: sc-358,914; Santa Cruz Biotechnology Inc.). DAB staining (CAS no: 7411-49-6; Santa Cruz Biotechnology Inc.) was used as a chromogenic reagent. The nuclei were stained using Mayer’s hematoxylin solution. Slides were observed with light microscopy.

### Statistical analysis

Data are expressed as mean ± SD. We used the student t-test to compare the differences between the two groups (Graphpad Prism V.8). P < 0.05 was considered significant.

## Results

### Decellularized bovine ovarian tissues efficiency

**Fig. 2:**
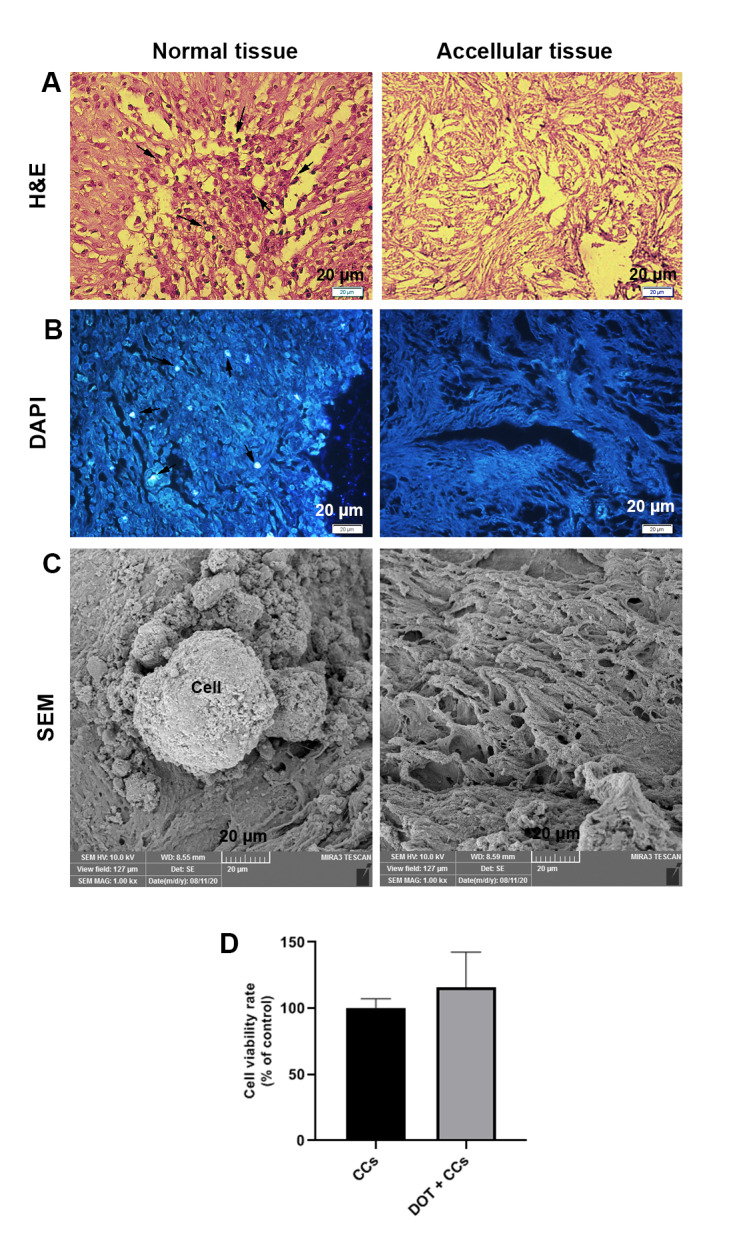
Confirmation of decellularization process via several analyses. H & E staining showed the lack of cells within the ovarian parenchyma after the decellularization procedure (**A**). DAPI staining exhibited a lack of cell nuclei in the decellularized ovarian tissue compared to the control group (**B**). SEM imaging indicated increased porosity after decellularization (**C**). Scale bar = 20 µM. Cell viability rate (percentage) normalized to control group with LDH assay, n = 8 (**D**). DOT = decellularized ovarian tissue. CCs = cumulus cells. Black arrows show the cell nucleus

The general features of normal and acellular tissues were evaluated using the H&E staining. Data revealed that the decellularized ovarian tissue lacks cell nuclei compared to normal tissue structure (Fig. 2 A). Besides, data showed naïve and intact microarchitecture in decellularized samples. The remnant of DNA content was monitored using DAPI staining. Data showed the lack of nuclei and DNA remnants in decellularized groups compared to the control samples (Fig. 2B). SEM imaging showed fibrous structure in ovarian tissue ECM after the decellularization procedure (Fig. 2 C). To be specific, data confirmed intact 3D ECM structure with normal interstitial tissue devoid of cells. PAS and Masson’s Trichrome staining were done to evaluate the levels of carbohydrate macromolecules and collagen fibers in ovarian tissue after acellularization. Data revealed a faint red-color appearance in tissue slides after PAS staining. Along with these changes, we noted the lack of blue-colored collagen fiber after the decellularization process (**Supplementary Fig. 1**). These features showed the efficiency of our protocol to yield acellular ovarian tissue.

### Cell expansion and survival

Bright-field imaging exhibited typical morphology for expanded HUVECs and CCs (**Supplementary Fig. 2**). To study whether the culture of CCs on decellularized scaffolds can affect the permeability of cell membranes, we measured supernatant LDH contents. LDH assay showed a non-significant increase of LDH content in the CCs group cultured on decellularized ovarian tissue after 7 days compared to the control acellular samples (p > 0.05; Fig. 2D). These data showed the lack of cytotoxicity in decellularized ovarian samples with prominent CC membrane integrity.

### In vivo transplantation

**Fig. 3:**
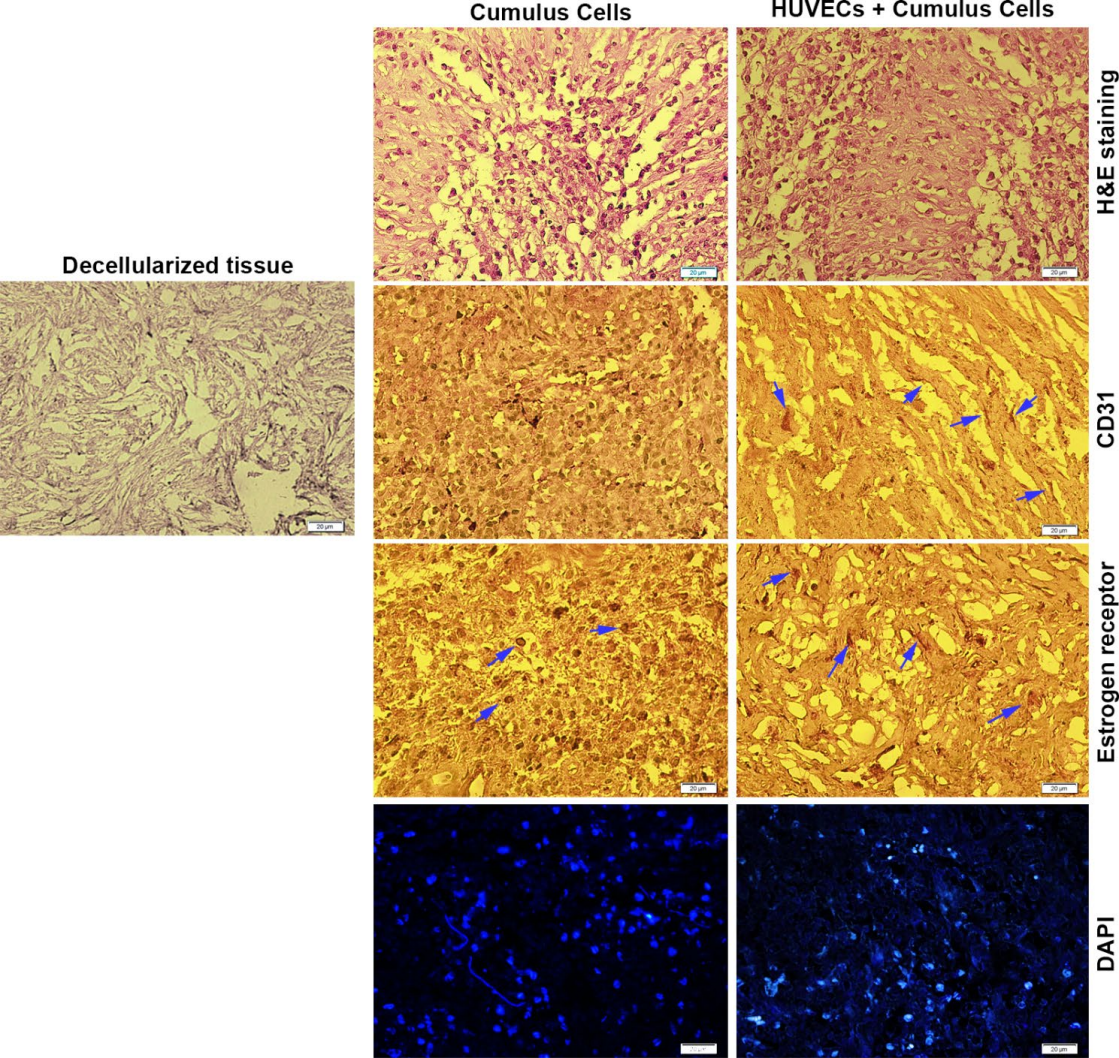
Recellularization process, CCs and HUVECs culture (passage 3), magnification = 40X (**A**), H & E, and DAPI staining following recellularization. IHC staining against CD31 and estrogen receptor antibodies (**B**). Arrows indicate the positive cells after staining. Scale bar = 20 µM

To analyze the histological changes of the recellularized ovarian tissue after subcutaneous transplantation in a rat model, H&E staining was performed. Histological analysis of transplants showed the existence of numerous cells and nuclei inside decellularized ovarian tissue 14 days after transplantation (Fig. 3). It seems that the combination of HUVECs and CCs resulted in the formation of dense ECM related to the CCs and control acellular groups. Besides, the cells in both groups (CCs and CCs + HUVECs) were evenly distributed within the decellularized parenchyma. These data exhibit an appropriate niche for cell migration and proliferation. IHC staining showed ER-positive cells in both CCs and CCs + HUVECs groups. Along with the statement, CD31 positive cells were indicated in CCs + HUVECs groups. These cells appeared a flattened morphology aligned with ECM fibers. We did not find CD31-positive cells in the CCs group (Fig. 3). These data showed that rat ECs cannot migrate into the deeper layer of decellularized ovarian tissue after transplantation. Therefore, the combination of HUVECs, as endothelial lineage, with CCs can support blood perfusion and active dynamic growth. Along with these data, DAPI staining revealed the existence of numerous nuclei in both groups inside decellularized niche 14 days after transplantation.

## Discussion

Here, we successfully developed decellularized ovarian tissue using different chemicals. Microscopic imaging confirmed the lack of nuclei and DNA remnants with naïve ECM structure which is consistent with previously published data [7]. Likewise, it was notified that the decellularized ovarian tissue lack cells within the parenchyma. Monitoring the slides using PAS and Masson’s trichrome staining revealed the reduction of protein and macromolecule carbohydrates. It has been shown that the existence of protein and macromolecule carbohydrates can lead to the promotion of immune cell activity and immune-rejection responses [15]. The reduction of these biomolecules in the decellularized tissue can increase the regenerative potential of transplanted grafts in the host tissue.

Along with these changes, cell-free ECM can be detected after the introduction of ovarian tissues to the decellularization protocol. The naive structure of ECM after decellularization should support a niche for the culture and expansion of specific cell types. The 7-day incubation period of CCs on prepared decellularized samples led to a close-to-normal survival rate. In this regard, non-significant differences were found related to supernatant LDH levels compared to the CCs cultured on the plastic surface. In most of the previously conducted experiments, only one cell type has been used for recellularization and in vivo assays [8, 16, 17]. Given the physiology of CCs associated with oocyte function and HUVECs with angiogenic capacity, the combination of both cells was used to mimic an in vivo-like condition. Besides, IHC revealed the existence of CD31 and ER-positive cells within the decellularized ovarian tissue after transplantation into rat subcutaneous, indicating the angiogenic capacity of HUVECs and phenotype acquisition of CCs.

We also indicated a more condensed ECM in CCs + HUVECs group compared to the CCs. The encapsulation of recellularized ovarian tissues inside alginate-gelatin hydrogel was done to provide a protective layer for limiting immune cell invasion, immunological responses, and further deformation of transplants in the subcutaneous layer [18, 19]. This strategy seems to diminish the likelihood of rejection. It has been shown that the combination of alginate-gelatin can provide a specific niche for a better regenerative outcome [20]. Previously, Nemati and colleagues approved the angiogenic capacity of HUVECs within the alginate-based hydrogel. They showed that the combination of alginate-gelatin is eligible to increase angiogenesis-related tyrosine kinases such as Tie-1, -2, VEGFR-1, and − 2 [11]. We also found that CCs efficiently expressed ER which correlates with the dynamic activity of these cells [21, 22]. It has been shown that the culture of cells on gelatin cross-linked matrices supports appropriate cell growth and survival by providing several motifs [23]. It seems that the addition of alginate-gelatin to decellularized ovarian tissue acts as a suitable biological microenvironment for ECs and CCs proliferation, migration, and activity [24]. This property is touted to be enhanced in the presence of bovine whole ovarian tissue protein extract. Like our data, it has been shown that ECM can facilitate regeneration and restoration of ovarian tissue function as a natural scaffold in decellularized three-dimensional microstructure [25]. Therefore, obtaining ECM using decellularization methods can be a logical approach for tissue engineering firstly, due to its natural source, and secondly, it is a relatively inexpensive circumstance compared to synthetic scaffolds [7]. We think that the culture of certain cell types such as CCs in combination with ECs on decellularized ovarian tissue can be a promising approach to mimicking an *in-vivo* condition. A load of crude protein extract is an efficient way to promote cell differentiation and phenotype acquisition in acellular matrix. Regenerative medicine and tissue engineering has emerged as a strategy for alternative treatment. Tissue engineering by decellularization and recellularization of ECM has the advantage for the development of customized medicine in the context of organ transplantation and reproductive disorders.

## Limitations

The current experiment faces some limitations that need further consideration. In this study, we did use the crude extract of ovarian tissue for cell culture purposes. The application of specific ECM components can efficiently reflect the regenerative impact of each substrate in a decellularized structure subjected to CCs and HUVECs.

## Electronic supplementary material

Supplementary Figure 1. Mason’s Trichrome (A) and Periodic acid-Schiff (B) staining for the detection of type I collagen fibers and macromolecule carbohydrates after ovarian tissue decellularization (A).

Supplementary Figure 2. Cumulus cells (CCs) and HUVECs at passage three. CCs appeared as flattened structures with diverse morphologies while HUVECs exhibited cobblestone shapes.

## Data Availability

All data generated or analyzed during this study are included in this published article.

## Abbreviations

DAB: 3, 3′-diaminobenzidine
DAPI: 4′, 6-diamidino-2-phenylindole
CCs: Bovine cumulus cells
CF-KRH: Calcium-free Krebs ringer HEPES buffer solution
COC: Cumulus-oocyte complex
ER: Estrogen receptor
ECM: Extracellular matrix
FBS: Fetal bovine serum
H&E: Hematoxylin and Eosin
HUVECs: Human umbilical vein endothelial cells
LDH: Lactate dehydrogenase
PBS: Phosphate-buffered saline
SDS: Sodium dodecyl sulfate
