# Supplementary figures and images for: Decellularized bovine ovarian niche restored the function of cumulus and endothelial cells
Source: BMC Res Notes. 2022 Nov 8;15:346. doi: 10.1186/s13104-022-06233-7 (PMC9644448; doi:10.1186/s13104-022-06233-7)

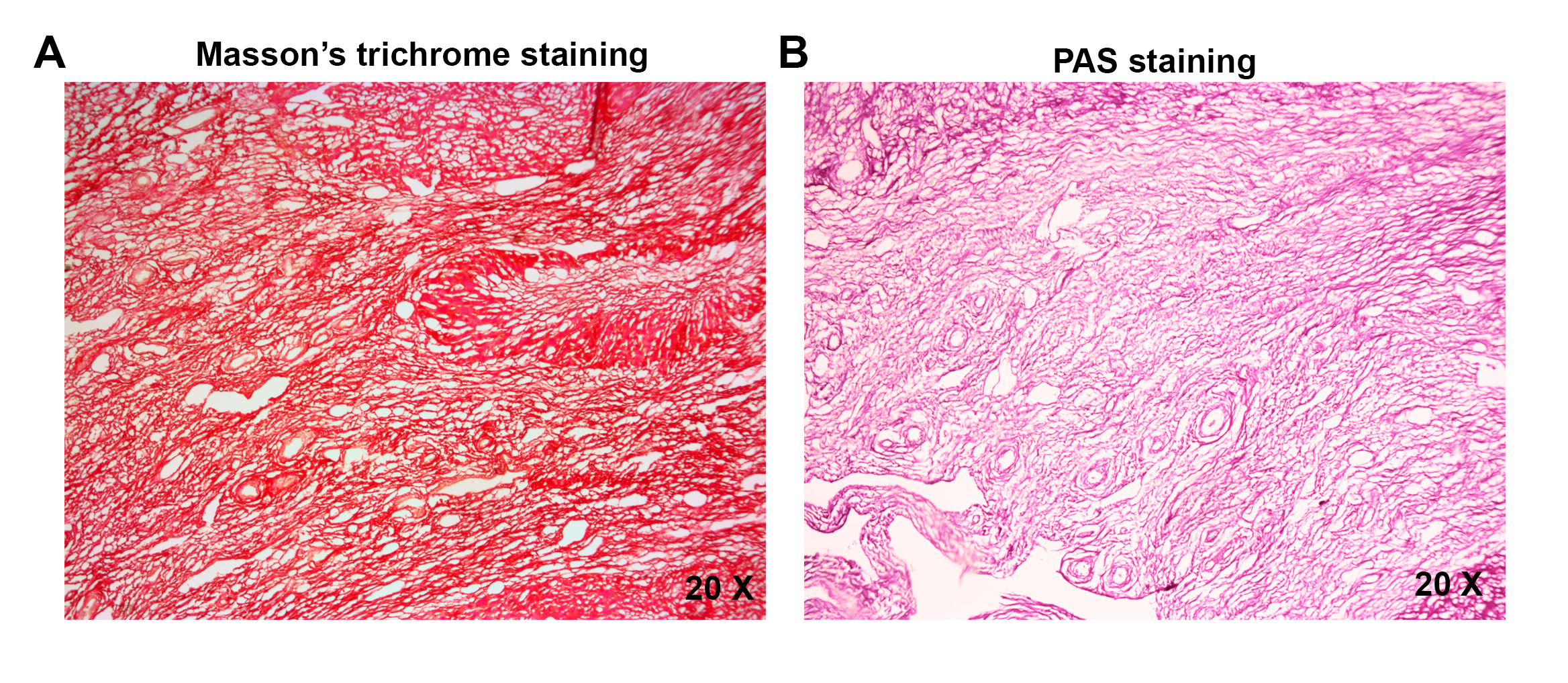

Supplement: Supplementary file 1 — Supplementary Figure 1. Mason’s Trichrome (A) and Periodic acid-Schiff (B) staining for the detection of type I collagen fibers and macromolecule carbohydrates after ovarian tissue decellularization (A). [file 13104_2022_6233_MOESM1_ESM.png]

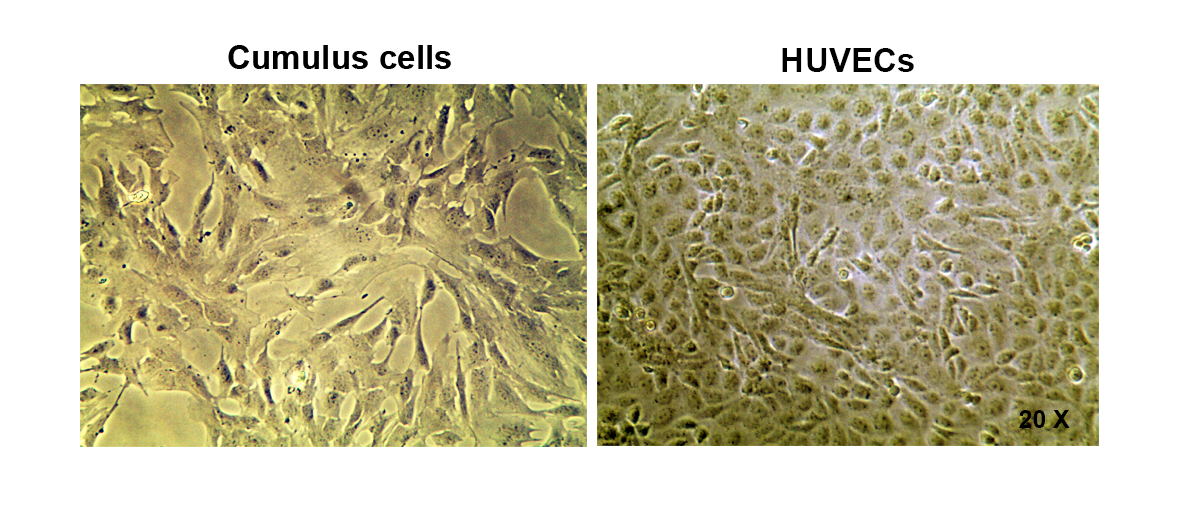

Supplement: Supplementary file 2 — Supplementary Figure 2. Cumulus cells (CCs) and HUVECs at passage three. CCs appeared as flattened structures with diverse morphologies while HUVECs exhibited cobblestone shapes. [file 13104_2022_6233_MOESM2_ESM.png]
